# Investigation of Flexible Arrayed Lactate Biosensor Based on Copper Doped Zinc Oxide Films Modified by Iron–Platinum Nanoparticles

**DOI:** 10.3390/polym13132062

**Published:** 2021-06-23

**Authors:** Yu-Hsun Nien, Zhi-Xuan Kang, Tzu-Yu Su, Chih-Sung Ho, Jung-Chuan Chou, Chih-Hsien Lai, Po-Yu Kuo, Tsu-Yang Lai, Zhe-Xin Dong, Yung-Yu Chen, Yu-Hao Huang

**Affiliations:** 1 Department of Chemical and Materials Engineering, National Yunlin University of Science and Technology, Yunlin 640, Taiwan; winnie19979@gmail.com (Z.-X.K.); szu19960517@gmail.com (T.-Y.S.); 2 Department of Chemical and Materials Engineering, Tunghai University, Taichung 407, Taiwan; csho@thu.edu.tw; 3 Department of Electronic Engineering, National Yunlin University of Science and Technology, Yunlin 640, Taiwan; choujc@yuntech.edu.tw (J.-C.C.); chlai@yuntech.edu.tw (C.-H.L.); kuopy@yuntech.edu.tw (P.-Y.K.); p12357585964@gmail.com (T.-Y.L.); wolverine010449@gmail.com (Z.-X.D.); m10813013@gmail.yuntech.edu.tw (Y.-Y.C.); m10813071@gemail.yuntech.edu.tw (Y.-H.H.)

**Keywords:** lactate detection, copper doped zinc oxide (CZO) sensing film, iron–platinum nanoparticles (FePt NPs), electrochemical sensor, non-enzymatic biosensor

## Abstract

Potentiometric biosensors based on flexible arrayed silver paste electrode and copper-doped zinc oxide sensing film modified by iron-platinum nanoparticles (FePt NPs) are designed and manufactured to detect lactate in human. The sensing film is made of copper-doped zinc oxide (CZO) by a radio frequency (RF) sputtering system, and then modified by iron-platinum nanoparticles (FePt NPs). The surface morphology of copper-doped zinc oxide (CZO) is analyzed by scanning electron microscope (SEM). FePt NPs are analyzed by X-ray diffraction (XRD) and Fourier transform infrared spectroscopy (FTIR). The average sensitivity, response time, and interference effect of the lactate biosensors are analyzed by voltage-time (V-T) measurement system. The electrochemical impedance is analyzed by electrochemical impedance spectroscopy (EIS). The average sensitivity and linearity over the concentration range 0.2–5 mM are 25.32 mV/mM and 0.977 mV/mM, respectively. The response time of the lactate biosensor is 16 s, with excellent selectivity.

## 1. Introduction

l-lactic acid (l-LA) is the most common cause of metabolic acidosis in the critical care setting, and has been associated with a large increase in mortality [1]. The definition of metabolic acidosis is a lactate level in the blood of higher than 5 mmol/L [2]. In the process of athlete training, vigorous exercise leads to acidosis due to the rapid increase of lactate, which causes the release of protons and the formation of acid salt sodium lactate.

The measurement of lactate level is very important, because it can aid in the diagnosis of different diseases. If the production rate of lactate is too high, it exceeds the proton buffer capacity of the cell, and results in the decrease of the pH in the cell. As acidosis causes damage to muscle cell membranes, the leakage of intracellular substances into the blood leads to a decline in body function and even multiple organ failure [3,4].

There are many methods for the determination of lactate such as polarimetry [5], gas chromatography (GC) [6], high performance liquid chromatography (HPLC) [7], and nuclear magnetic resonance (NMR) [8]. However, these methods are expensive and require complicated sample preparation and trained personnel. For the above reasons, a simple and fast method needs to be developed. A chemical sensor is proposed, and its sensing method is simple, fast, and has high sensitivity. Electrochemical sensors are proposed. The biosensors use enzymes as the sensing element in order to accurately sense the analyte, because enzymes only react with specific substances. Commonly used enzymes for lactate sensors are lactate oxidase (LOX) [9] and lactate dehydrogenase (LDH) [10].

Electrochemical sensors based on redox active enzymes (LOX and LDH enzymes) are widely used due to their high sensitivity and selectivity. However, this type of sensor has some disadvantages, such as high cost, low temperature storage of enzymes, environmental sensitivity, and a complicated manufacturing process. Therefore, replacing enzymatic sensors is our goal. Currently, non-enzymatic lactic acid sensors with low cost, high sensitivity, fast response time, and repeatability are being studied [11], such as biomimetic lactate imprinted smart polymers [12] and various nanostructured metal oxides such nickel oxide (NiO) [13] and zinc oxide (ZnO) [14].

A non-enzymatic lactic acid sensor has the problem of being susceptible to interference and low sensitivity, so that we use Cu-doped ZnO to provide a more stable sensing film. Doped ZnO film causes significant changes in resistance, higher sensitivity, and a lower operating temperature [15]. The most commonly used metal dopants in ZnO-based systems are Al, Co, Cu, Ga, Ni, Sn, etc. Due to its similar electronic shell structure, Cu has many physical and chemical properties similar to Zn [16]. It has been shown that substituting copper into the ZnO lattice can improve its sensing characteristics [17].

The development of artificial enzymes for nanomaterials has grown rapidly. In contrast to natural enzymes, enzyme mimics have been found to have better flexibility, high stability under harsh conditions, high toughness and versatility, and low cost. FePt can produce electrochemical reactions and is an ideal catalyst for slow redox processes. It can also act as an active component for effectively accelerating the electron transfer between the electrode and analyte, which leads to a rapid current response and a reduced overpotential for electrochemical reactions [18]. In this study, we have added FePt NPs to accelerate the redox process of lactic acid and the electron transfer between the electrode and the analyte, for enhancing the sensor’s sensing ability.

## 2. Materials and Methods

### 2.1. Materials

Polyethylene terephthalate (PET) was obtained from Zencatec Corporation, Taoyuan City, Taiwan. Silver conductive paste was procured from Advanced Electronic Material Inc., Tainan City, Taiwan. Copper-doped zinc oxide (CZO) target with Cu 3% ZnO 97% was bought from Ultimate Materials Technology Co., Ltd., Ping Tung City, Taiwan. Epoxy (a class of thermosetting polymers with product no. JA643) was procured from (Sil-More Industrial, Ltd., New Taipei City, Taiwan). Iron acetylacetonate and oleyl amine was obtained from Acros Organics (Ward Hill, MA, USA). Platinum acetylacetonate was bought from Alfa Aesar (Haverhill, MA, USA). Oleic acid was procured from Showa Chemical (Tokyo, Japan).

### 2.2. Fabrication of Oil-Soluble FePt NPs

**Figure 1:**
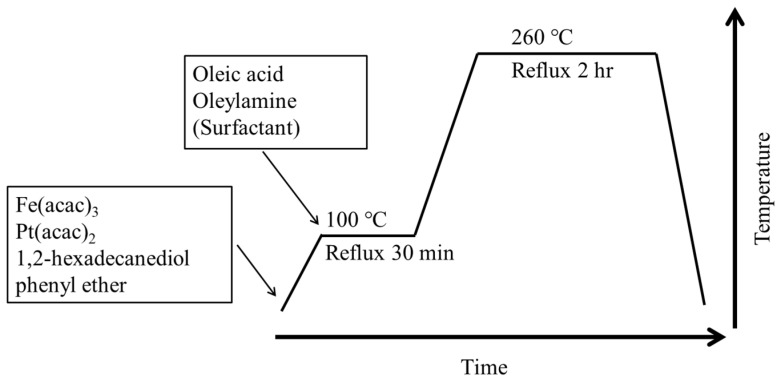
Flow chart of iron–platinum nanoparticles (FePt NPs) synthesis.

**Figure 2:**
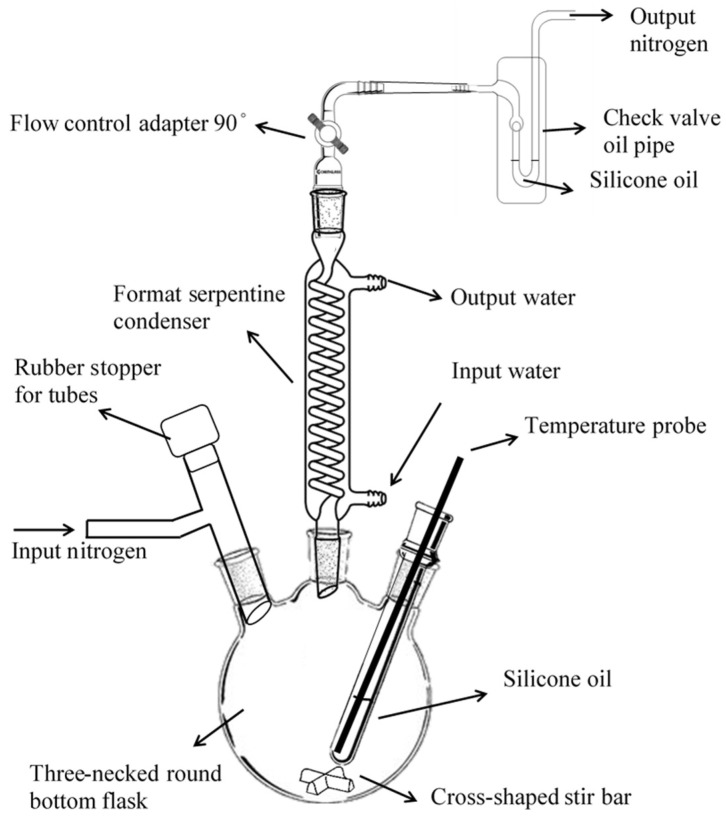
Iron–platinum nanoparticles (FePt NPs) synthesis device diagram.

FePt NPs were synthesized via the decomposition of iron acetylacetonate and reduction of platinum acetylacetonate in the presence of oleic acid and oleyl amine stabilizers. It was synthesized under anaerobic conditions [19]. Iron acetylacetonate (1 mmol), platinum acetylacetonate (0.25 mmol), 1,2-hexadecanediol (0.75 mmol), and 30 mL phenyl ether were put into a three-necked flask. The mixture was dissolved by the ultrasonic vibrator. It was heated from 5 °C to 100 °C at a heating rate of 5 °C/min. Oleic acid (0.5 mmol) and oleylamine (1 mmol) were quickly added to the three-necked flask with a syringe. The mixture was heated from 100 °C to 260 °C at a heating rate of 7 °C/min, followed by reflux for 2 h, and then naturally cooled to room temperature under a nitrogen stream. Ethanol was added to precipitate FePt particles and remove impurities. The flow chart and synthesis device diagram of FePt NPs are shown in Figure 1 and Figure 2.

### 2.3. Fabrication of Water-Soluble FePt NPs

The oil-soluble FePt NPs exchange surface bonds through thioglycolic acid [20]. The oil-soluble FePt NPs were dissolved in chloroform at the concentration of 1 mg (FePt NPs)/mL. FePt NPs were dissolved using an ultrasonic oscillator. The 1 mM thioglycolic acid was added in FePt/chloroform at the concentration of 1 mg (FePt NPs)/mL. The mixture was stirred vigorously for 2 h. We remove the chloroform and add ethanol to wash the excess thioglycolic acid, for repeating three times. The water-soluble FePt NPs were dried in a vacuum oven at 60 °C for 1 day.

### 2.4. Fabrication of Flexible Lactate Biosensors Based on FePt NPs/CZO Sensing Films

**Figure 3:**
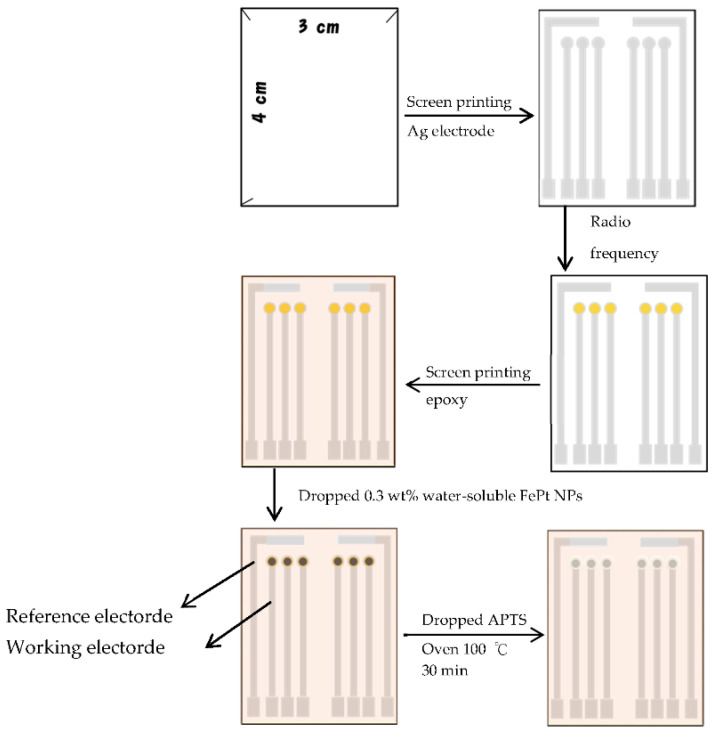
The diagram of the fabrication of the lactate biosensor.

An Ag electrode was printed by screen printing technology, and CZO film was sputtered on the Ag electrode by radio frequency (RF) sputtering. The CZO film was prepared by Cu-ZnO alloy to deposit the CZO film on the Ag electrode under the conditions of chamber pressure of 3 × 10^−6^ torr, argon flow of 9 sccm, oxygen flow of 1 sccm, RF power of 120 W, and sputtering time of 20 min. Epoxy was printed on the lactate biosensor through the screen-printing machine for electrode packing. The 0.3 wt% water-soluble FePt NPs were dropped on the sensing window of the CZO film. The APTS (3-Aminopropyl triethoxysilane) was used as a protective layer and dropped on the CZO film modified by FePt NPs. The lactate biosensor was heated in an oven at 100 °C for 30 min. A diagram of the step is shown in Figure 3.

### 2.5. The V-T Measurement System

**Figure 4:**
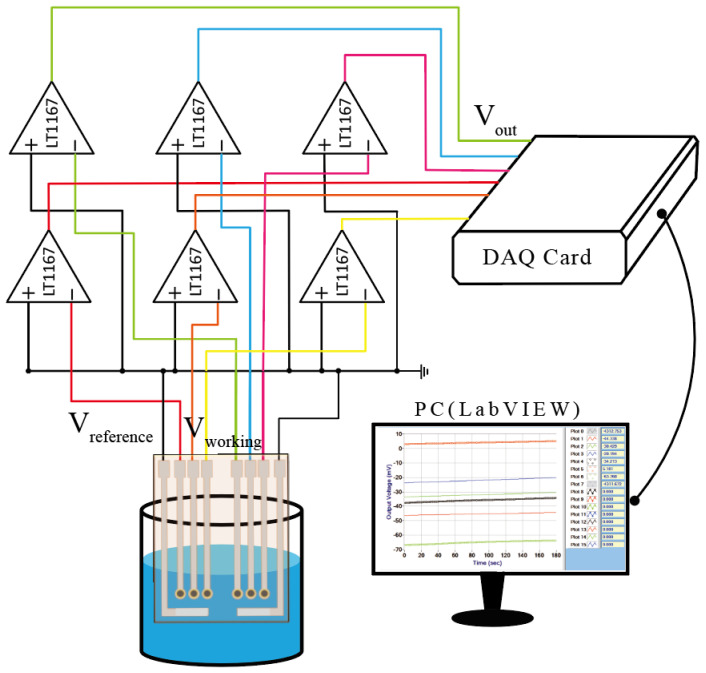
The V-T measurement system.

The sensing characteristics of the voltage flexible lactate biosensor are detected by the voltage measurement system. The measurement system is shown in Figure 4. The lactate biosensor is immersed in a lactate solution. According to electrooxidation, lactate is converted into pyruvic acid and hydrogen peroxide (H_2_O_2_) × H_2_O_2_ releases H^+^ and converts to HO_2_. Then, HO_2_ releases electrons to produce O_2_ and H^+^. Due to these released electrons and H^+^, the electrochemical response of the FePt NPs/CZO sensor to detect lactate is realized. The response voltage of the electrode is sent to the instrumentation amplifier (LT1167) to stabilize the response voltage and reduce the noise from the ground. We provide the instrumentation amplifier (LT1167) with a working voltage of ±3 V. The output voltage of the instrumentation amplifier is transmitted to the data acquisition card (DAQ card), and the output voltage is converted to a digital signal. The limit resolution of the DAQ card is 10.4 μV. The data is transferred to a computer and analyzed using LabVIEW software.

### 2.6. Description of the Conditions Used for the Characterization of CZO Film Modified by FePt NPs

FE-SEM used in this study is from JEOL (model JSM-6701F). The magnification is 100,000, and the field energy is 15.0 kV. XRD used in this study is from Rigaku (model MiniFlex II). The scan range is from 30° to 80° with a scanning speed of 2° min^−1^. FTIR used in this study is from Perkin Elmer (model Spectrum One). The wavelength range is from 4000 cm^−1^ to 400 cm^−1^ with a resolution of 1–4 cm^−1^.

## 3. Results

### 3.1. The SEM and EDS of the CZO Sensing Film

**Figure 5:**
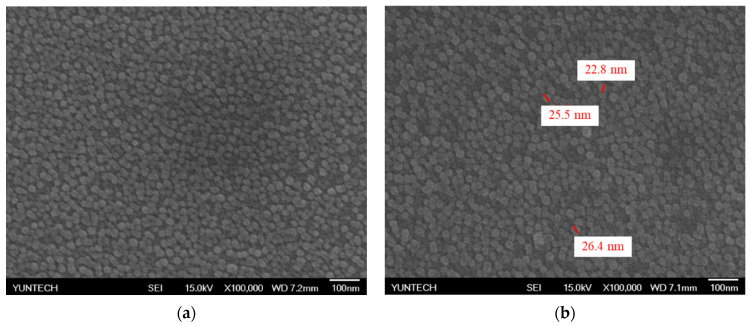
The FE-SEM images of (**a**) the surface morphology of copper-doped zinc oxide (CZO) film and (**b**) the particle size of CZO film.

The CZO film is deposited on the silicon substrate through a sputtering system. The surface of the CZO film is observed by FE-SEM as shown in Figure 5a. According to Figure 5a, the particle size of CZO is similar, and the film is deposited on the sensor window uniformly without agglomeration of CZO particles. This provides a stable sensing platform for lactic acid sensing. According to Figure 5b, the particle size of CZO is measured by SEM. The particle size of CZO is 24.9 ± 1.8 nm. The element content is analyzed by energy dispersive spectrometer (EDS) as shown in Table 1. Table 1 shows the weight ratio and atomic ratio of the elements of the CZO film on the silicon substrate. The ratio of the target material we used is Cu:ZnO = 3:97, and the EDS result accords with the ratio of the target material.

### 3.2. X-ray Diffraction of the FePt NPs

**Figure 6:**
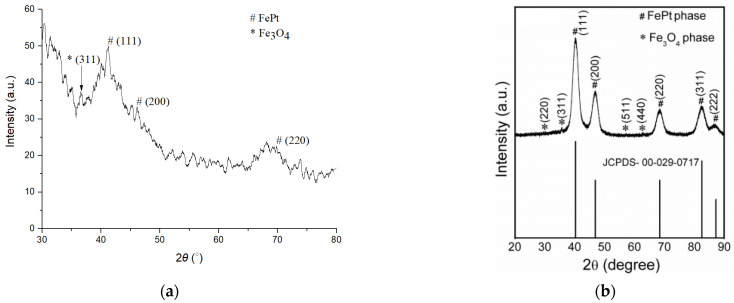
The X-ray diffraction (XRD) patterns of (**a**) the FePt NPs and (**b**) the JCPDS #-29-0717 (fcc-FePt).

The X-ray diffraction (XRD) pattern of the FePt NPs is shown in Figure 6a. The diffraction peaks at 41.7° (111), 46.6° (200), and 70.0° (220) are the main diffraction peaks of FePt. The diffraction peak at 36.8° (311) is Fe_3_O_4_ that is formed when oleic acid is used as an active agent in the process of FePt synthesis. Fe(acac)_3_ is reduced to Fe_3_O_4_ when 1,2-hexadecanediol is used as a reducing agent. The small diffraction peak of (311) means that FePt has a complete reaction during the synthesis process, and no oxygen enters the device during the synthesis process. The JCPDS #-29-0717 (fcc-FePt) [21] is shown in Figure 6b.

### 3.3. Fourier Transform Infrared Spectroscopy (FTIR) of the FePt NPs

**Figure 7:**
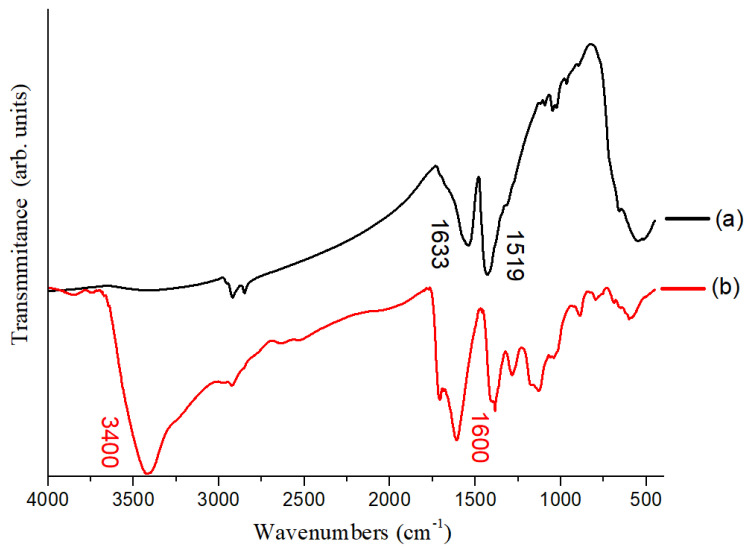
The Fourier-transform infrared spectroscopy (FTIR) patterns of (**a**) water soluble FePt NPs and (**b**) oil soluble FePt NPs.

The mechanism of FePt phase inversion is that the thiol group (–SH) is bonded to the surface of FePt NPs, and the end of the functional group is connected to the carboxyl group (–COOH). In order to confirm whether the FePt NPs ligand exchange has been successful, we use Fourier transform infrared spectroscopy (FTIR) to measure the functional groups of FePt NPs before and after ligand exchange. From the part of the oil soluble FePt NPs in Figure 7, we can observe the peak with –COO– functional group at 1519 cm^−1^, and the peak with –NH_2_– functional group at 1633 cm^−1^. These two functional groups are the functional groups produced by surfactants and FePt NPs. The oil soluble FePt NPs can be uniformly dispersed. From the part of the water soluble FePt NPs in Figure 7, there is a signal of –OH– functional group at 3400 cm^−1^. There is a signal of C=O functional group at 1600 cm^−1^. From the presence of C=O and –OH-functional groups, FePt NPs have successful ligand exchange, and –OH-functional groups can make FePt NPs uniformly disperse in the solution.

### 3.4. The Sensing Characteristics of Flexible Lactate Biosensor Based on FePt NPs/CZO Sensing Films

**Figure 8:**
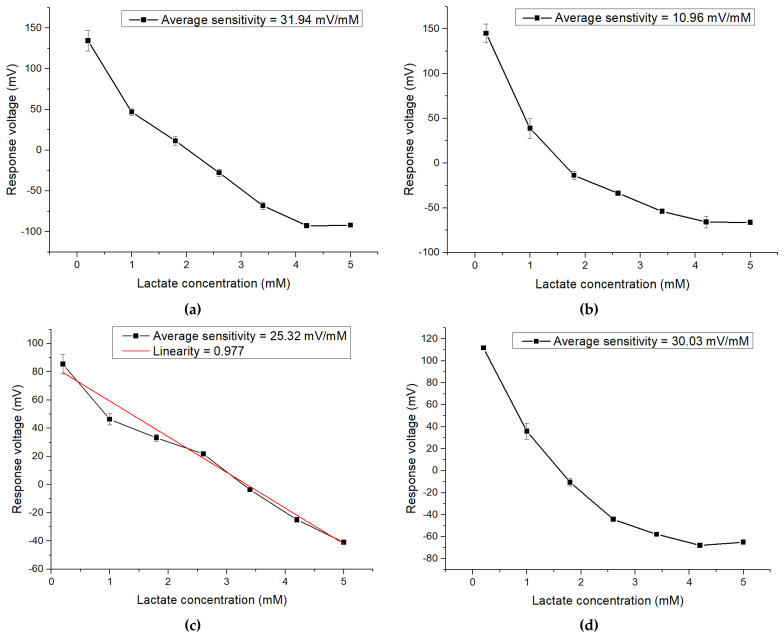
The average sensitivity and linearity of lactate biosensor modified by (**a**) 0.05 wt% FePt NPs, (**b**) 0.1 wt% FePt NPs, (**c**) 0.3 wt% FePt NPs, and (**d**) 0.5 wt% FePt NPs.

In this work, four different amounts of FePt NPs are added to modify the potentiometric flexible arrayed lactate biosensor based on a CZO sensing membrane. The biosensor is immersed in the PBS solution containing lactate, and their concentrations are 0.2 mM, 1 mM, 1.8 mM, 2.6 mM, 3.4 mM, 4.2 mM and 5 mM, respectively, and measured by the V-T measurement system and the response voltages. The 0.05 wt% FePt NPs and 0.1 wt% FePt NPs showed no change in the measurement voltage at 4.2 mM and 5 mM. It means that the amount of FePt NPs is not enough to catalyze lactate, meaning that different response voltages cannot be measured. The linearity of lactate sensors modified with 0.5 wt% FePt NPs is poor, because too many FePt NPs block the sensing window. According to Figure 8, the samples with 0.05 wt% and 0.1 wt% FePt NPs have no linear relationship. The the sample with 0.3 wt% FePt NPs has the best average sensitivity and linearity. The average sensitivity and linearity of the lactate sensors modified with 0.3 wt% FePt NPs are shown in Figure 8c. The average sensitivity and linearity are 25.32 mV/mM and 0.977, respectively, as shown in Table 2.

### 3.5. Response Time

**Figure 9:**
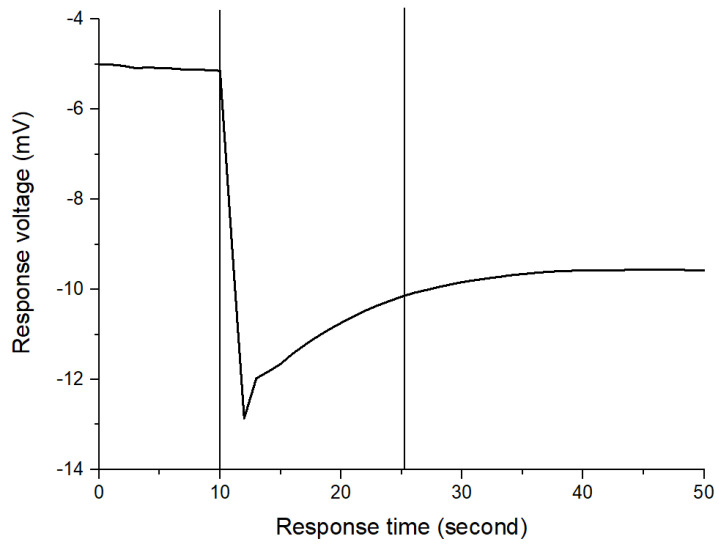
The curves of response time for flexible lactate biosensor based on FePt NPs/CZO sensing films.

The response time is defined as the time required for the output voltage to change from its initial state to 95% of the steady state after immersing the sensor in the test solution [22,23]. In this study, the lactate biosensor was immersed in the PBS solution. When the biosensor was stable, 2.5 mL of 100 mM lactate was dropped into the PBS solution. The shorter the response time, the faster the biosensor responses to the analyte. The response time of the biosensor based on FePt NPs/CZO sensing films is 16 s. The response time of the biosensor based on FePt NPs/CZO sensing films is shown in Figure 9.

### 3.6. Interference Effect

**Figure 10:**
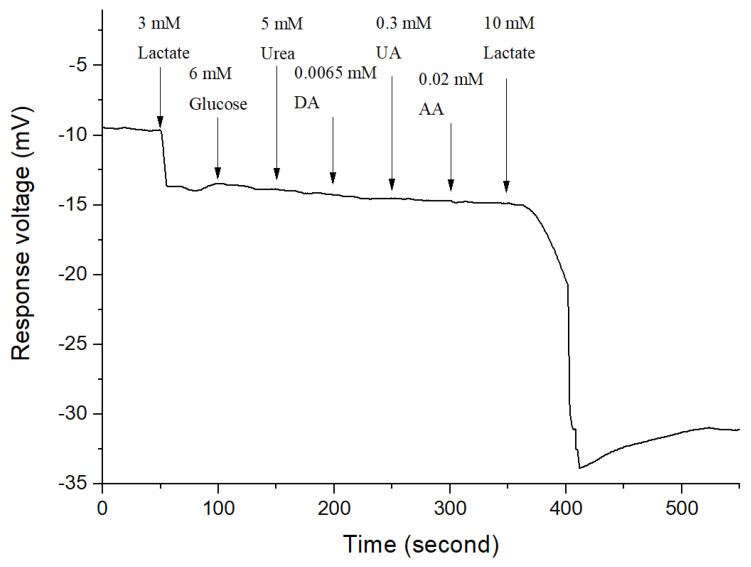
The interferences effect of the flexible lactate biosensor based on CZO film/FePt NPs after adding 6.00 mM glucose, 5.00 mM urea, 0.06 mM dopamine (DA), 0.30 mM uric acid (UA), 0.02 mM ascorbic acid (AA), and 10.0 mM lactate.

In order to evaluate the selectivity of the lactate biosensor based on CZO film/FePt NPs, the interference effect experiment was performed. First, the lactate biosensor was immersed in PBS solution, and then 3 mM lactate, 6 mM glucose, 5 mM urea, 0.065 mM dopamine (DA), 0.3 mM uric acid (UA), and 0.02 mM ascorbic acid (AA) were added every 50 s, respectively. During the lactate measurement, these substances were considered the main interference factors [24]. The lactate concentration was increased up to 10 mM to investigate the selectivity of the lactate biosensor. The result of the interference experiment is shown in Figure 10. The concentration of different interferences is depicted in Table 3.

### 3.7. The Analysis of Electrochemical Impedance Spectroscopy of Lactate Biosensor Based on Different Sensing Membrane

**Figure 11:**
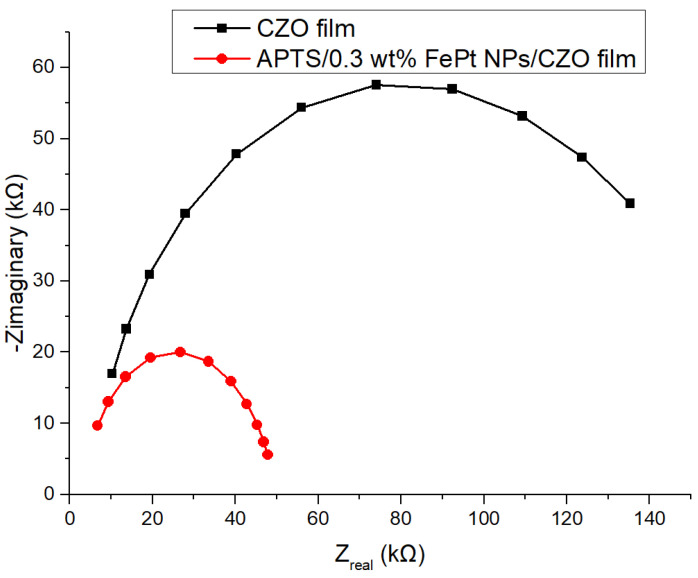
The curves of EIS analysis for flexible lactate biosensor based on FePt NPs/CZO sensing films, and equivalent circuit (inset).

Electrochemical impedance spectroscopy represents an effective method for detecting resistance. Adding nanomaterials to the surface of electrodes or semiconductors changes the capacitance and interface electron transfer resistance of conductive or semiconductor electrodes. The charge transfer resistances of FePt NPs/CZO are investigated and the results are shown in Figure 11. The impedance value of CZO films is 96,520 Ω, and the impedance value of APTS/FePt NPs/CZO films is 23,398 Ω.

We compare the results of this research with the previous enzyme formula of the research group. According to Table 4, the lactate measurement range of this study is relatively large, but the average sensitivity is relatively low. The reason is that enzymes can grab more lactic acid. The disadvantage is that it is difficult to store and complicated to prepare. The manufacturing process in this study is relatively simple, and it can be stored at room temperature and has a large measurement range, which improves the performance of this lactate biosensor.

## 4. Conclusions

In this work, a flexible array lactate biosensor using FePt NPs/CZO sensing film has been proposed. The CZO film has been analyzed by an energy dispersion spectrometer. The surface morphology and the element contents have been confirmed by SEM and EDS analysis. XRD results show that the synthesized FePt has the main diffraction peaks of FePt. FTIR confirms that the FePt is water soluble after ligand exchange. The obvious –OH peak indicates that the –OH functional group can make FePt NPs uniformly disperse in the solution. Through the voltage-time (V-T) measurement systems, a variety of sensing characteristics are analyzed, including sensitivity, linearity, response time, and interference effects. The lactate biosensor based on FePt NPs/CZO sensing films have a sensitivity of 25.32 mV/mM, linearity of 0.977, and response time of 16 s. The EIS analysis is applied to measure the flexible arrayed lactate biosensors based on FePt NPs/CZO, CZO sensing films. According to the EIS analysis, FePt NPs/CZO sensing films have minimal charge transfer resistance and excellent charge transfer capability.

## Figures and Tables

**Table 1:** The element contents of copper-doped zinc oxide (CZO) film.

Element Series				
	Unnormalized Weight %	Normalized Weight %	Atomic %	Error
	(wt. %)	(wt. %)	(at. %)	(wt. %)
Copper	0.87	3.73	2.77	0.2
Zinc	19.63	83.81	60.47	1.8
Oxygen	2.92	12.46	36.76	1.4
Total	23.43	100.00	100.00	

**Table 2:** Comparison of the average sensitivity and linearity based on different concentrations of FePt NPs.

Concentration of FePt NPs (wt%)	Average Sensitivity (mV/mM)	Linearity
0.00	12.76	Nonlinear
0.05	31.94	Nonlinear
0.10	10.96	Nonlinear
0.30	25.32	0.977
0.50	30.03	Nonlinear

**Table 3:** The concentration of different interference substances.

Interference Substances	Concentration (mM)
Glucose	5.00
Urea	5.00
Dopamine (DA)	0.06
Uric Acid (UA)	0.30
Ascorbic Acid (AA)	0.02

**Table 4:** Comparisons of linear range, average sensitivities, and linearity of potentiometric lactate biosensors with various sensing membranes.

Sensing Members	Linear Range (mM)	Average Sensitivity (mV/mM)	Linearity	Reference
LDH NAD^+^-IGZO	0.3–3	56.09	0.998	[25]
LDH NAD^+^-MBs/GPTS/GO/NiO	0.2–3	45.40	0.992	[26]
MBs-LDHNAD^+^-GO/IGZO	0.2–3	69.08	0.997	[27]
γ-APTS/0.3%FePt NPs/CZO	0.2–5	25.31	0.977	This study

## Data Availability

Not applicable.
